# Outdoor Smoking Areas: Does the Science Support a Ban?

**DOI:** 10.1289/ehp.121-a229

**Published:** 2013-07-01

**Authors:** Carol Potera

**Affiliations:** Carol Potera, based in Montana, has written for *EHP* since 1996. She also writes for *Microbe*, *Genetic Engineering News*, and the *American Journal of Nursing*.

Inhaling secondhand cigarette smoke (SHS), also known as passive smoking, can cause cancer and respiratory and cardiovascular disease.^1^ Indoor smoking is banned at many public places and worksites; at others, smoking areas have been moved outdoors. But is keeping cigarette smoke outdoors enough to dissipate the health risks associated with SHS exposure? A review in this issue of *EHP* explores the current state of knowledge about outdoor SHS exposure.^2^

“Outdoor SHS is an emerging topic in the tobacco control community,” says review coauthor Esteve Fernández, an epidemiologist at the Institut Català d’Oncologia–ICO in Barcelona, Spain. Surveys indicate that public support for banning outdoor smoking has increased in recent years,^3^ although some opponents argue that such bans are unsustainable, unduly restrictive, and unsupported by the evidence to date.^4^^,^^5^ To impose outdoor smoking laws, tobacco control advocates need “evidence-based results from valid and representative epidemiological studies about levels of SHS in different outdoor areas,” says Fernández.

For the current review Fernández and colleagues analyzed data from 18 scientific papers published between 2005 and 2012, which measured SHS exposure at outdoor settings in Europe, the United States, Canada, Australia, and New Zealand. Sites included hospitality venues (e.g., restaurants and bars), airports, parks, streets, entrances to buildings, and college campuses.^2^

In most of the studies reviewed, the main marker for SHS was fine particulate matter (PM_2.5_). Measured average levels of PM_2.5_ ranged from 8.32 μg/m^3^ to 124 μg/m^3^ at outdoor hospitality venues where smokers were present, and from 4.6 μg/m^3^ to 17.8 μg/m^3^ at other outdoor settings. Individual point measurements exceeded 1,000 μg/m^3^ in some cases. Densely packed smokers, partially enclosed outdoor areas, low wind speeds, and closeness to people smoking all contributed to high levels of outdoor SHS. Smoke-free indoor settings near outdoor smoking areas also had elevated PM_2.5_ levels, with mean concentrations ranging from 4 μg/m^3^ to 120.51 μg/m^3^.

Measured levels exceeded the median level for irritation from secondhand smoke PM_2.5_ reported for brief exposures.^6^ Most studies detected outdoor concentrations of PM_2.5_ exceeding 10 μg/m^3^, the annual outdoor average that the World Health Organization sets as the lowest cutoff at which lung cancer and cardiopulmonary deaths are likely to increase^7^—a particular concern for chronically exposed hospitality workers. “Although outdoor SHS levels are more transient than indoor levels, and can quickly drop to background levels in the absence of active smoking,” the authors wrote, “potential health effects of these exposures merit consideration and need to be further studied.”

**Figure:**
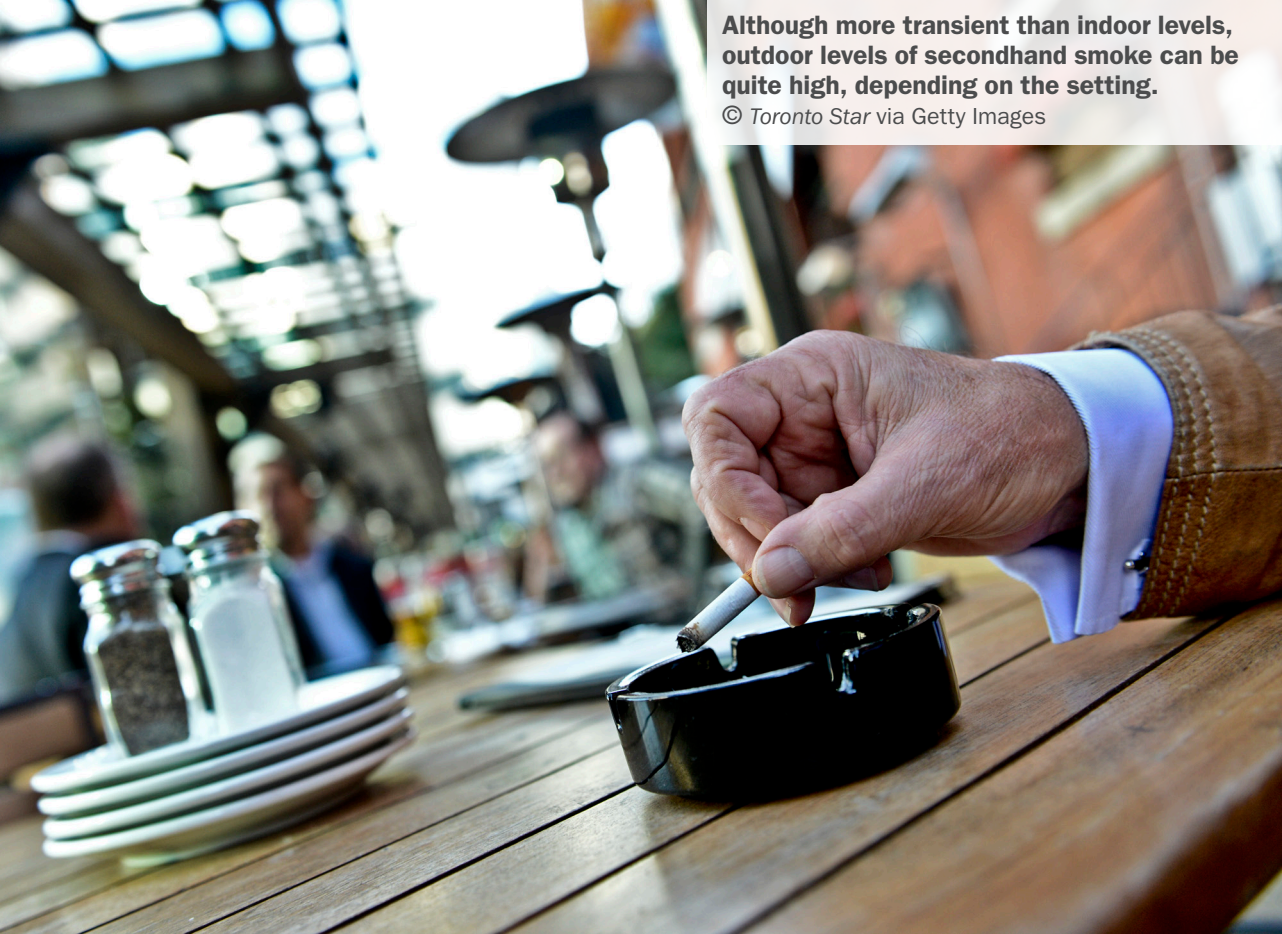
Although more transient than indoor levels, outdoor levels of secondhand smoke can be quite high, depending on the setting. © Toronto Star via Getty Images

The review shows that “depending on the microenvironment, you can get very high levels of secondhand smoke outdoors. A cigarette is a point source of outdoor pollution,” says Stanton Glantz, director of the Center for Tobacco Control Research and Education at the University of California, San Francisco. Fernández’s compilation of data scattered across different journals “will be useful to policy makers. The evidence points to banning outdoor SHS where smokers congregate,” Glantz says.

The compiled data also highlight the need for better standardized methods in future studies. PM_2.5_, although cheap and easy to measure, is a common traffic pollutant and not specific to SHS. More precise and sensitive markers such as salivary cotinine (a metabolic by-product of nicotine) better reflect personal exposure to SHS.^2^ An ideal study would combine both environmental markers such as airborne nicotine and biological markers such as cotinine in saliva. “Such studies, although more complicated to implement, would be of extreme relevance,” says Fernández.

SHS contains more than 7,000 chemicals, including about 70 known and probable carcinogens, as well as toxicants and irritants.^1^ In the United States, an estimated 46,000 premature deaths from heart disease and 3,400 lung cancer deaths in nonsmokers are caused by SHS exposure yearly.^1^ Gene microarray scans of cells lining the small airways suggest there are no safe levels of SHS exposure. One study showed that even very low exposure was associated with changes in gene expression that may reflect early smoking-induced damage, potentially setting the stage for lung disease and cancer.^8^
